# The landscape of MET alterations in non-small cell lung cancer in Southeastern China: a real-world study

**DOI:** 10.1186/s12885-026-16211-y

**Published:** 2026-05-26

**Authors:** Sisi Zheng, Ziyi Zuo, Rixu Lin, Xiaoxiao Zhang, Lujie Huang, Mengsi Cai, Yifan Ye, Dan Yao, Xiaoying Huang

**Affiliations:** 1 Pulmonary Division, the First Affiliated Hospital of Wenzhou Medical University, Wenzhou Key Laboratory of Interdisciplinary and Translational Medicine, Wenzhou Key Laboratory of Heart and Lung, Wenzhou, Zhejiang 325035 China; 2 Pathology Department, the First Affiliated Hospital of Wenzhou Medical University, Wenzhou, Zhejiang 325035 China

**Keywords:** MET, NSCLC, MET-TKI, Survival analysis, Real-world

## Abstract

**Background:**

MET is a key driver in NSCLC yet comprehensive, multi-subtype real-world data in China are lacking. Methods: Real-world data were collected from MET-altered NSCLC patients diagnosed at a comprehensive hospital in southeastern China (July 2021–December 2023). Four subgroups were analyzed: MET IHC-Positive, MET Amplification, MET Exon 14 skipping, and MET Other Mutations. Demographics, risk factors, comorbidities, pathology, co-mutation, and treatment patterns were compared. With 2-year follow-up, survival outcomes and MET-TKI benefit were analyzed.

**Results:**

Among 574 patients: MET IHC-Pos (81.4%), MET-Amp (11.0%), MET-Ex14 (4.70%), and Others (2.96%). Patients were predominantly elderly, male, with > 40% smoking history and > 35% hypertension. Pathological specimens were mainly obtained via bronchoscopy or percutaneous lung puncture. Adenocarcinoma dominated all subtypes (> 70%), increasing with IHC intensity in MET IHC-Pos (*p* < 0.05). MET-Amp showed the highest advanced stage (87.3%), bone (36.5%) and brain metastasis (17.5%). TP53 was the most common co-mutation (20.7%–25.0%). Targeted therapy predominated (Line 1st: 54.6%, Line 2nd: 38.9%), followed by chemotherapy plus immunotherapy. The 24-month OS was lower in MET-Amp (44.4%) and MET-Ex14 (48.1%) than in MET IHC-Pos (57.2%) and Others (58.8%). The 24-month OS (MET-TKI treated vs. untreated) was: 69.2% vs. 38.5% (MET IHC-Pos 2 + /3 +), 71.4% vs. 25.0% (MET-Ex14), and 47.4% vs. 42.9% (MET-Amp).

**Conclusions:**

MET alterations occur predominantly in the elderly, males, and smokers. MET-Ex14 shows driver-dependent characteristics with clear MET-TKI benefit. MET-Amp presents the most aggressive phenotype. MET IHC-Pos predominates (> 80%) with heterogeneity; the 2 + /3 + may benefit from MET-TKI. These findings inform subtype-based management of MET-altered NSCLC in southeastern China.

**Supplementary Information:**

The online version contains supplementary material available at 10.1186/s12885-026-16211-y.

## Introduction

Mesenchymal-epithelial transition factor (MET) is a key driver gene in non-small cell lung cancer (NSCLC), encoding the hepatocyte growth factor receptor and participating in cell proliferation, migration, invasion, and angiogenesis [1]. MET alterations encompass various forms, including MET Exon 14 skipping, MET Amplification, and MET Overexpression [2–4]. Regardless of subtype, all are associated with poor prognosis, with median survival typically under 24 months [5, 6].

In China, the approval of savolitinib in 2021 [7] has promoted MET alteration testing to the initial diagnosis stage. However, systematic understanding of the comprehensive landscape of MET alterations remains inadequate. First, existing clinical trials have primarily focused on MET Exon 14 skipping, the subtype with the most clearly defined therapeutic target [1]. Data on MET amplification and MET overexpression remain limited. Second, previous studies have largely concentrated on single dimensions, such as molecular characteristics or treatment efficacy. Comprehensive real-world studies integrating demographics, risk factors, comorbidities, pathological characteristics, co-mutation patterns, treatment strategies, and survival outcomes remain scarce. This gap limits the development of precision therapeutic approaches.

More importantly, the prevalence of MET alterations differs significantly between Chinese and Western populations. The incidence of MET Exon 14 skipping (0.9–1.7%) [8, 9] and MET Overexpression (13.7–63.7%) in Chinese populations is lower than that in Western populations (2–4% and 15–75%) [10, 11]. These disparities in racial background, healthcare resource accessibility, and MET detection strategies limit the generalizability of Western findings to Chinese patients [12]. Southeastern China offers a favorable setting for this study, given its higher economic development, greater molecular testing availability, and better access to novel therapies.

Accordingly, this study retrospectively analyzed MET-altered NSCLC patients diagnosed at a large comprehensive medical center in southeastern China between July 2021 and December 2023 (30 months). Patients were classified into four subtypes: MET IHC-Positive, MET Amplification, MET Exon 14 Skipping, and MET Other Mutations. The study aims to: (1) describe demographic characteristics, risk factor exposure, and comorbidity burden; (2) characterize pathological features, metastatic and co-mutation patterns; (3) analyze treatment patterns (including MET-TKI utilization); (4) evaluate survival outcomes and MET-TKI treatment impact across MET-altered subtypes, providing evidence for precision stratified management of MET-altered NSCLC in southeastern China.

## Methods

### Study population

This study consecutively enrolled patients with MET-altered NSCLC diagnosed at the First Affiliated Hospital of Wenzhou Medical University between July 1, 2021, and December 31, 2023. All patients had complete 2-year follow-up records. The study was conducted in accordance with the Declaration of Helsinki and was approved by the Ethics Committee of the First Affiliated Hospital of Wenzhou Medical University (Approval Number: KY2024-R069). As a retrospective study, the requirement for informed consent was waived.

### Definition of MET alterations

MET alterations were detected in tissue or liquid samples using at least one of the following methods: RT-qPCR (Real-Time Quantitative Polymerase Chain Reaction), IHC (Immunohistochemistry), NGS (Next-Generation Sequencing), or FISH (Fluorescence In Situ Hybridization). Patients were classified into four subtypes as follows:

1. MET IHC-Positive: MET protein expression was assessed using the SP44 monoclonal antibody (Ventana, Roche Diagnostics). Positivity was defined as ≥ 50% of tumor cells showing positive staining (intensity 1 + to 3 +) and further stratified into IHC 1 +, IHC 2 +, and IHC 3 + subgroups. The scoring criteria were: 1 + (weak to moderate staining in ≥ 50% of tumor cells), 2 + (moderate to strong staining in ≥ 50% of tumor cells, but < 50% with strong staining), and 3 + (strong staining in ≥ 50% of tumor cells).
2. MET Amplification: determined by FISH according to UCCC (University of Colorado Cancer Center) criteria, defined as meeting any of the following: MET/CEP7 ratio ≥ 2.0, or MET gene copy number ≥ 15 in ≥ 10% of cells, or high polysomy (≥ 4 MET copies in ≥ 40% of cells), or presence of signal clusters or atypically large and bright MET signals in ≥ 10% of cells.
3. MET Exon 14 Skipping: confirmed by RT-qPCR or NGS.
4. MET Other Mutations: included rare exon variants detected by NGS. No MET fusion was identified in this cohort.

For patients with multiple MET alterations detected, hierarchical classification was applied based on clinical relevance priority: MET Exon 14 Skipping > MET Amplification > MET IHC-Positive > MET Other Mutations.

### Data collection and endpoints

Data on patient demographics, risk factor exposure, comorbidities, pathological characteristics, co-mutation profiles, and treatment information were collected. Tumor staging was based on the American Joint Committee on Cancer (AJCC) 9th edition TNM staging system, with early stage defined as I–IIIA and advanced stage as IIIB–IV. MET-TKI treatment included one of below: Savolitinib, Glumetinib, Capmatinib, Tepotinib, or Bozitinib.

The primary endpoint was overall survival (OS), defined as the time from initial MET alteration detection to death from any cause or last follow-up. All patients had complete 24-month follow-up, with data censored on December 31, 2025.

### Statistical analysis

Continuous variables were presented as median (interquartile range, IQR) and categorical variables were summarized as frequencies (percentages). Group comparisons were performed using the chi-square test or Fisher's exact test. Post-hoc pairwise comparisons were conducted with Bonferroni correction for multiple testing.

Survival time was measured from MET alteration detection to death or last follow-up, with survival censored at 24 months for patients still alive. Survival curves were generated using the Kaplan–Meier method and compared between groups using the log-rank test.

To control for confounding factors, propensity score matching (PSM) was performed using 1:2 nearest neighbor matching with a caliper value. Matching quality was assessed using standardized mean difference (SMD), with SMD < 0.1 considered indicative of well-balanced covariates. All statistical analyses were performed using R software (version 4.5.1), and statistical significance was defined as *p* < 0.05.

## Results

### Classification and baseline characteristics

**Fig. 1:**
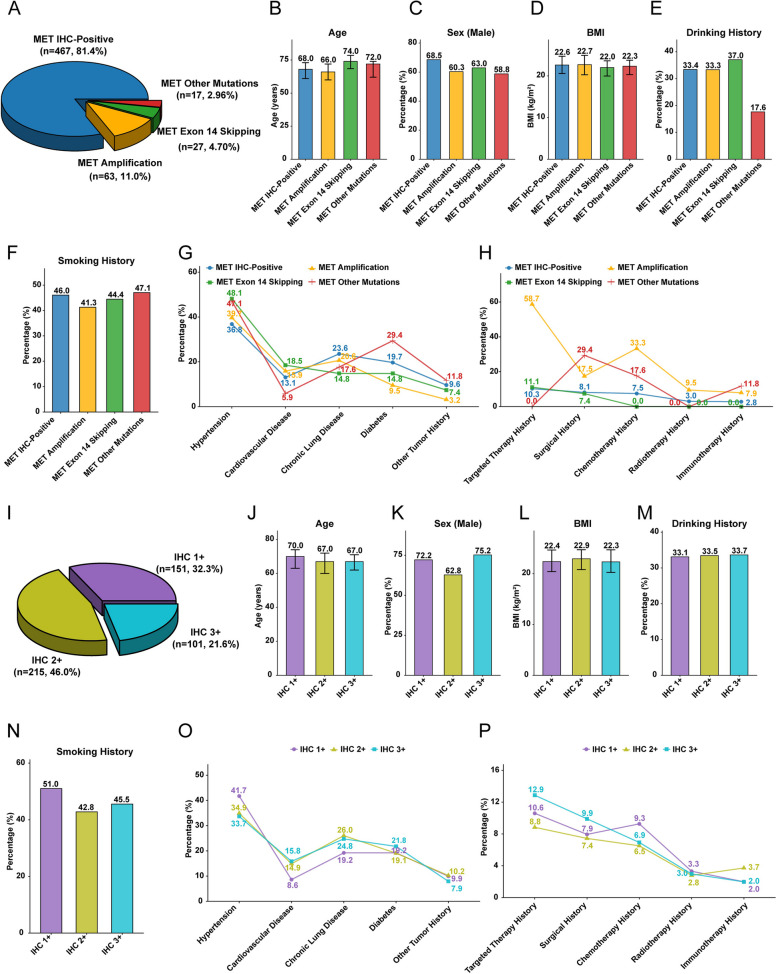
Baseline characteristics across MET alteration subtypes. (**A**) Distribution of MET alteration subtypes (*n* = 574). (B-H) Age (**B**), gender (**C**), BMI (**D**), drinking history (**E**), smoking history (**F**), comorbidity distribution (**G**), and prior treatment patterns (**H**) among four MET alteration subtypes. (**I**) MET IHC-Positive subtype composition (*n* = 467). (J-P) Age (**J**), gender (**K**), BMI (**L**), drinking history (**M**), smoking history (**N**), comorbidity distribution (**O**), and prior treatment patterns (**P**) among three IHC intensity groups. Age and BMI are presented as median (IQR), and categorical variables as percentages

A total of 574 patients with MET-altered NSCLC diagnosed at a large comprehensive hospital in southeastern China between July 1, 2021, and December 31, 2023, were enrolled. Patients were classified into four groups: MET IHC-Positive (*n* = 467, 81.4%), MET Amplification (*n* = 63, 11.0%), MET Exon 14 Skipping (*n* = 27, 4.70%), and MET Other Mutations (*n* = 17, 2.96%) (Fig. 1A).

Baseline characteristics are presented in Tables 1 and 2. Overall, patients across the four groups were predominantly elderly and male, with median age ranging from 66.0 to 74.0 years, male proportion from 58.8% to 68.5%. BMI ranged from 22.0 to 22.7 kg/m^2^ across subtypes. More than 40% of patients had a smoking history, and over 35% had hypertension.

**Table 1:** Clinical and pathological characteristics across MET alteration subtypes

Patients' characteristics	MET IHC-Positive (*n* = 467)	MET Amplification (*n* = 63)	MET Exon 14 Skipping (*n* = 27)	MET Other Mutations (*n* = 17)
Age(years), median (IQR)	68 (61–73)	66 (60–72)	74 (68–78)	72 (62–74)
Gender, n (%)				
Male	320 (68.5%)	38 (60.3%)	17 (63.0%)	10 (58.8%)
Female	147 (31.5%)	25 (39.7%)	10 (37.0%)	7 (41.2%)
BMI (kg/m2), median (IQR)	22.59 (20.57–24.69)	22.70 (20.26–24.91)	22.00 (19.95–23.60)	22.31 (20.31–23.74)
Smoking History, n (%)	215 (46.0%)	26 (41.3%)	12 (44.4%)	8 (47.1%)
Drinking History, n (%)	156 (33.4%)	21 (33.3%)	10 (37.0%)	3 (17.6%)
Hypertension, n (%)	172 (36.8%)	25 (39.7%)	13 (48.1%)	8 (47.1%)
Diabetes, n (%)	92 (19.7%)	6 (9.5%)	4 (14.8%)	5 (29.4%)
Chronic Lung Disease, n (%)	110 (23.6%)	13 (20.6%)	4 (14.8%)	3 (17.6%)
Cardiovascular Disease, n (%)	61 (13.1%)	10 (15.9%)	5 (18.5%)	1 (5.9%)
Other Tumor History, n (%)	45 (9.6%)	2 (3.2%)	2 (7.4%)	2 (11.8%)
Surgical History, n (%)	38 (8.1%)	11 (17.5%)	2 (7.4%)	5 (29.4%)
Chemotherapy History, n (%)	35 (7.5%)	21 (33.3%)	0 (0.0%)	3 (17.6%)
Radiotherapy History, n (%)	14 (3.0%)	6 (9.5%)	0 (0.0%)	0 (0.0%)
Immunotherapy History, n (%)	13 (2.8%)	5 (7.9%)	0 (0.0%)	2 (11.8%)
Targeted Therapy History, n (%)	48 (10.3%)	37 (58.7%)	3 (11.1%)	0 (0.0%)
Histological type, n (%)				
Squamous cell carcinoma	103 (22.1%)	8 (12.7%)	2 (7.4%)	2 (11.8%)
Adenocarcinoma	333 (71.3%)	53 (84.1%)	20 (74.1%)	14 (82.4%)
Adenosquamous carcinoma	8 (1.7%)	1 (1.6%)	3 (11.1%)	0 (0.0%)
Large cell carcinoma	4 (0.9%)	1 (1.6%)	1 (3.7%)	0 (0.0%)
Other	19 (4.1%)	0 (0.0%)	1 (3.7%)	1 (5.9%)
TNM stage category, n (%)				
Early stage (I-IIIA)	149 (31.9%)	8 (12.7%)	7 (25.9%)	6 (35.3%)
Advanced stage (IIIB-IV)	318 (68.1%)	55 (87.3%)	20 (74.1%)	11 (64.7%)
Bone metastasis, n (%)	141 (30.2%)	23 (36.5%)	7 (25.9%)	3 (17.6%)
Lung metastasis, n (%)	69 (14.8%)	11 (17.5%)	5 (18.5%)	3 (17.6%)
Brain metastasis, n (%)	61 (13.1%)	11 (17.5%)	2 (7.4%)	0 (0.0%)

**Table 2:** Clinical and pathological characteristics according to IHC intensity in MET IHC-Positive patients

Patients' characteristics	IHC 1 + (*n* = 151)	IHC 2 + (*n* = 215)	IHC 3 + (*n* = 101)
Age(years), median (IQR)	70 (63–74)	67 (60–72)	67 (62–71)
Gender, n (%)			
Male	109 (72.2%)	135 (62.8%)	76 (75.2%)
Female	42 (27.8%)	80 (37.2%)	25 (24.8%)
BMI (kg/m2), median (IQR)	22.36 (20.38–24.63)	22.90 (20.79–24.70)	22.30 (20.22–24.67)
Smoking History, n (%)	77 (51.0%)	92 (42.8%)	46 (45.5%)
Drinking History, n (%)	50 (33.1%)	72 (33.5%)	34 (33.7%)
Hypertension, n (%)	63 (41.7%)	75 (34.9%)	34 (33.7%)
Diabetes, n (%)	29 (19.2%)	41 (19.1%)	22 (21.8%)
Chronic Lung Disease, n (%)	29 (19.2%)	56 (26.0%)	25 (24.8%)
Cardiovascular Disease, n (%)	13 (8.6%)	32 (14.9%)	16 (15.8%)
Other Tumor History, n (%)	15 (9.9%)	22 (10.2%)	8 (7.9%)
Surgical History, n (%)	12 (7.9%)	16 (7.4%)	10 (9.9%)
Chemotherapy History, n (%)	14 (9.3%)	14 (6.5%)	7 (6.9%)
Radiotherapy History, n (%)	5 (3.3%)	6 (2.8%)	3 (3.0%)
Immunotherapy History, n (%)	3 (2.0%)	8 (3.7%)	2 (2.0%)
Targeted Therapy History, n (%)	16 (10.6%)	19 (8.8%)	13 (12.9%)
Histological type, n (%)			
Squamous cell carcinoma	50 (33.1%)	44 (20.5%)	9 (8.9%)
Adenocarcinoma	87 (57.6%)	158 (73.5%)	88 (87.1%)
Adenosquamous carcinoma	2 (1.3%)	6 (2.8%)	0 (0.0%)
Large cell carcinoma	1 (0.7%)	1 (0.5%)	2 (2.0%)
Other	11 (7.3%)	6 (2.8%)	2 (2.0%)
TNM stage category, n (%)			
Early stage (I-II)	47 (31.1%)	73 (34.0%)	29 (28.7%)
Advanced stage (III-IV)	104 (68.9%)	142 (66.0%)	72 (71.3%)
Bone metastasis, n (%)	48 (31.8%)	64 (29.8%)	29 (28.7%)
Lung metastasis, n (%)	21 (13.9%)	21 (9.8%)	27 (26.7%)
Brain metastasis, n (%)	17 (11.3%)	23 (10.7%)	21 (20.8%)

Some variations were observed among subtypes. The MET Exon 14 Skipping group had the highest median age (74.0 years), while MET Amplification had the lowest (66.0 years). The MET Other Mutations group had a lower drinking history rate (17.6%) compared to other subtypes (MET IHC-Positive: 33.4%, MET Amplification: 33.3%, MET Exon14 Skipping: 37.0%) (Fig. 1B-G).

The MET Amplification group had higher rates of prior anticancer treatment across most modalities, particularly targeted therapy (58.7%), chemotherapy (33.3%) and radiotherapy (9.5%), compared to other subtypes (Fig. 1H).

To further characterize heterogeneity within MET IHC-Positive, patients were stratified into IHC 1 + (*n* = 151, 32.3%), IHC 2 + (*n* = 215, 46.0%), and IHC 3 + (*n* = 101, 21.6%) by staining intensity (Fig. 1I). Baseline characteristics were generally comparable across IHC intensity subgroups. Patients in IHC 2 +/3 + were slightly younger than IHC 1 + (both 67.0 vs. 70.0 years) (Fig. 1J-P) (Table 2).

### Pathological characteristics and metastatic patterns

#### Specimen acquisition and histological types

**Fig. 2:**
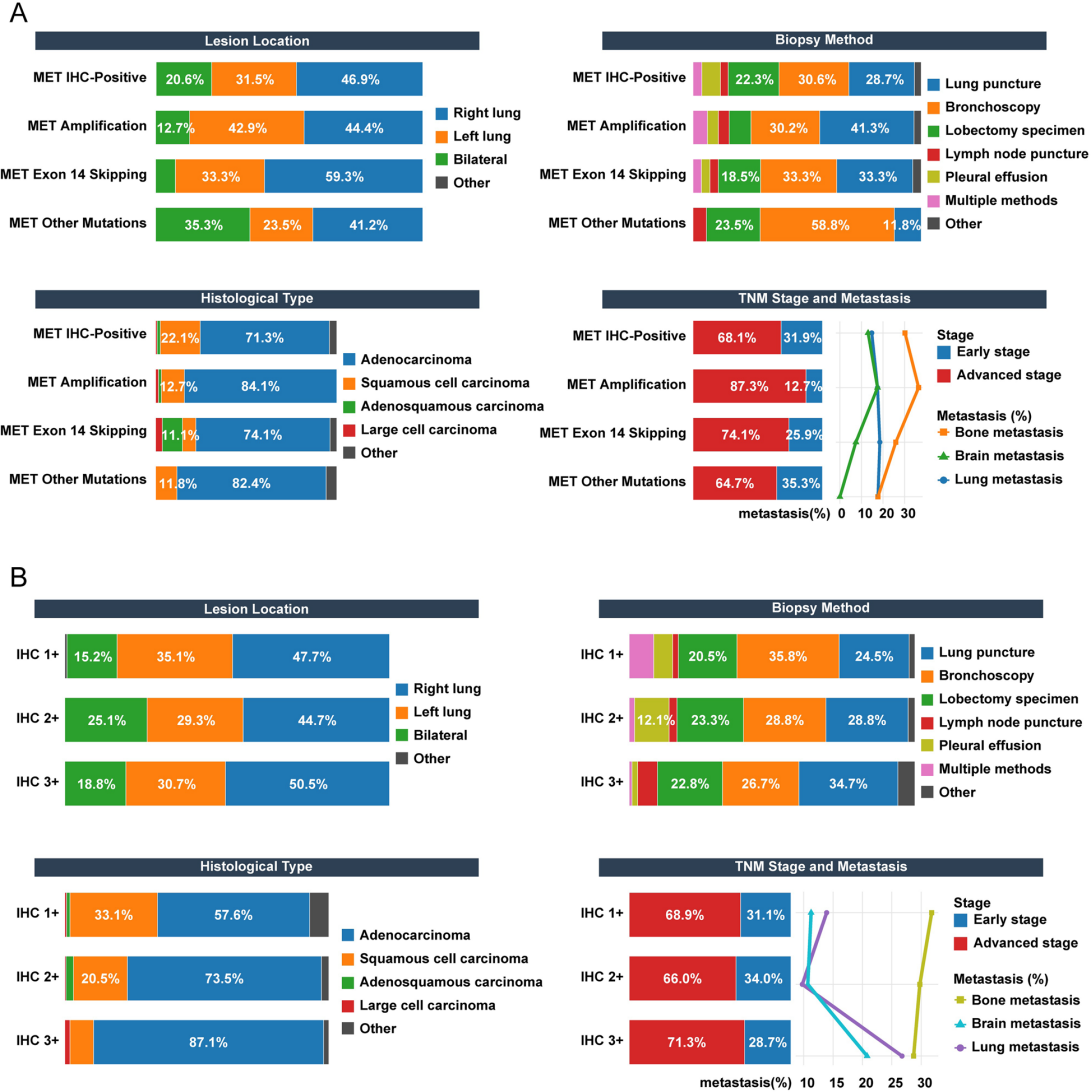
Pathological characteristics and metastatic patterns across MET alteration subtypes. **A** Distribution of lesion location, biopsy methods, histological types, TNM stage, and metastatic patterns across MET alteration subtypes. **B** Corresponding characteristics among three IHC intensity groups. Stacked bar charts show percentage composition of categorical variables. Line graphs show incidence rates of bone, brain, and lung metastasis

Patients showed similarities in lesion distribution and sampling methods across MET alteration subtypes. Tumor lesions were predominantly located in the right lung (41.2% to 59.3%). Bronchoscopic biopsy (30.2%–58.8%) and percutaneous lung puncture biopsy (11.8%–41.3%) were the most common specimen acquisition methods. Lobectomy was more frequently performed in MET IHC-Positive (22.3%) and MET Other Mutations (23.5%) groups. Regarding histological types, adenocarcinoma was the most predominant, accounting for 71.3%–84.1% (Fig. 2A) (Table 1, Table S1).

#### Cancer staging and metastasis

Patients across subtypes were mainly at advanced stage (IIIB–IV, 64.7%–87.3%). The MET Amplification group had the highest proportion of advanced-stage (87.3%), while MET Other Mutations had the lowest (64.7%). For metastatic patterns, the MET Amplification group showed higher rates of bone metastasis (36.5%) and brain metastasis (17.5%) than other subgroups (Fig. 2A) (Table 1).

#### Stratified characteristics according to IHC intensity

Within the MET IHC-Positive subgroup, cancer characteristics were generally similar with some differences in metastatic patterns and histological composition.

The IHC 3 + group had higher rates of brain (20.8%) and lung metastasis (26.7%) than IHC 1 + (11.3% and 13.9%) and IHC 2 + (10.7% and 9.8%) (Fig. 2B) (Table 2, Table S2). Additionally, IHC staining intensity was significantly associated with histological type. The proportion of adenocarcinoma showed an increasing trend with staining intensity (57.6% in IHC 1 +, 73.5% in IHC 2 +, 87.1% in IHC 3 +; chi-square = 26.7, *P* < 0.001). All pairwise comparisons were statistically significant (Table S3).

### Co-mutation patterns

**Fig. 3:**
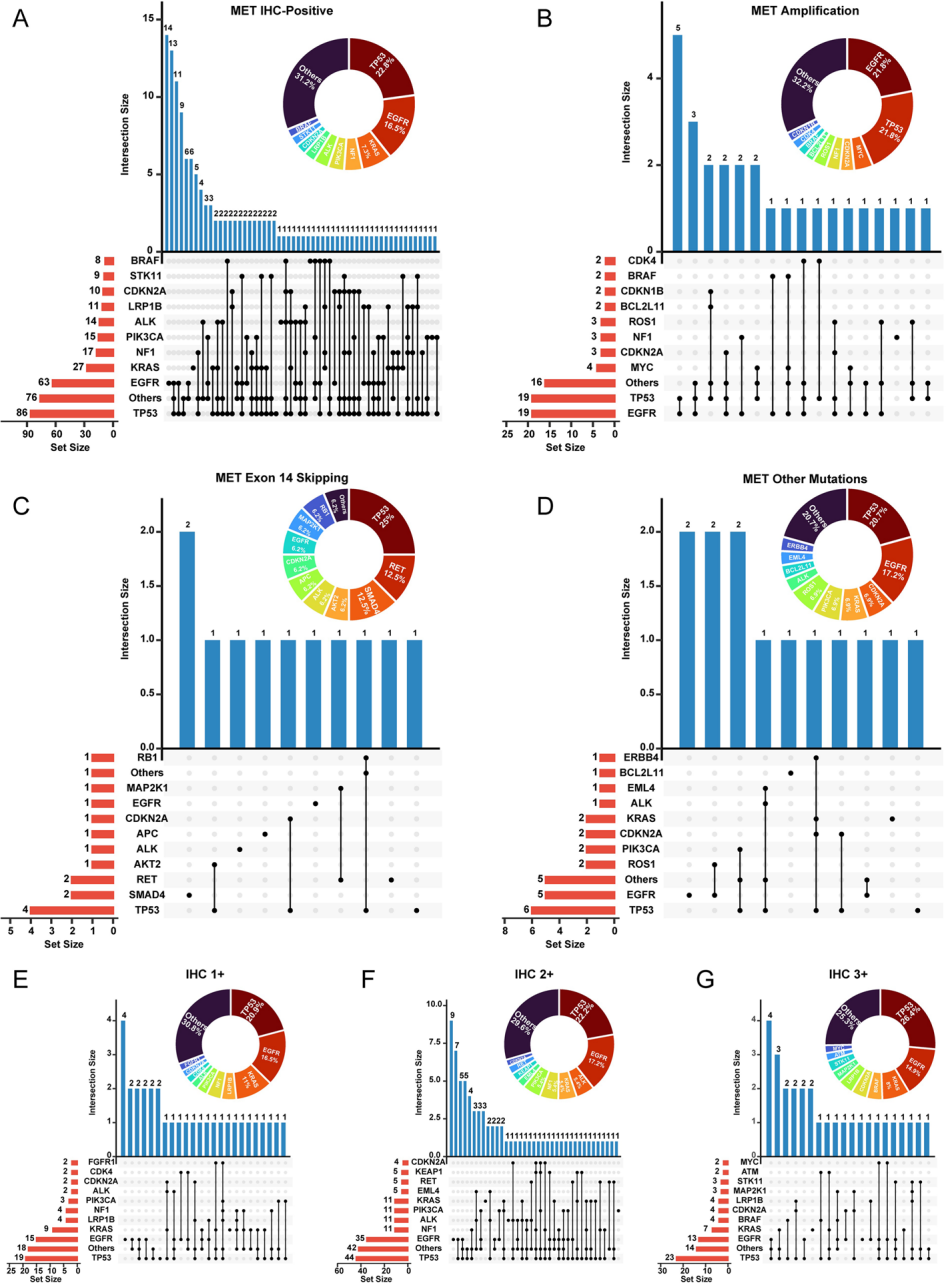
Co-mutation patterns across MET alteration subtypes. **A**-**D** UpSet plots showing gene mutation frequency and co-mutation patterns in MET IHC-Positive (**A**), MET Amplification (**B**), MET Exon 14 Skipping (**C**), and MET Other Mutation (**D**). Ring charts show the proportion of gene mutations. **E**–**G** UpSet plots and ring charts for the three IHC intensity groups: IHC 1 + (**E**), IHC 2 + (**F**), and IHC 3 + (**G**). In the UpSet plots, blue vertical bars represent co-mutation frequencies, and red horizontal bars indicate individual gene mutation frequencies. Only the top 10 genes by mutation frequency are displayed while remaining genes are categorized as Others

Among the patients included in the analysis, a total of 186 cases were detected with gene mutations other than MET. TP53 was the most common co-mutation across all MET alteration subtypes (20.7%–25.0%). EGFR was frequently observed in MET Amplification (21.8%), MET Other Mutations (17.2%), and MET IHC-Positive (16.5%) groups (Fig. 3A-D).

Besides, the combined EGFR-TP53 mutation pattern was prominent in the MET IHC-Positive and MET Amplification groups. In contrast, the MET Exon 14 Skipping group showed a dispersed co-mutation spectrum without a dominant pattern.

Further analysis revealed that co-mutation spectra were similar across IHC intensity subgroups. TP53 (20.9%–26.4%) and EGFR (14.9%–17.2%) remained the predominant co-mutated genes, with the combined EGFR-TP53 pattern persisting across all subgroups. Additionally, KRAS accounted for a certain proportion in each subgroup (11.0% in IHC 1 +, 5.4% in IHC 2 +, and 8.0% in IHC 3 +) (Fig. 3E-G).

### Treatment patterns

#### Overall MET treatment regimens: first-line vs. subsequent lines

**Fig. 4:**
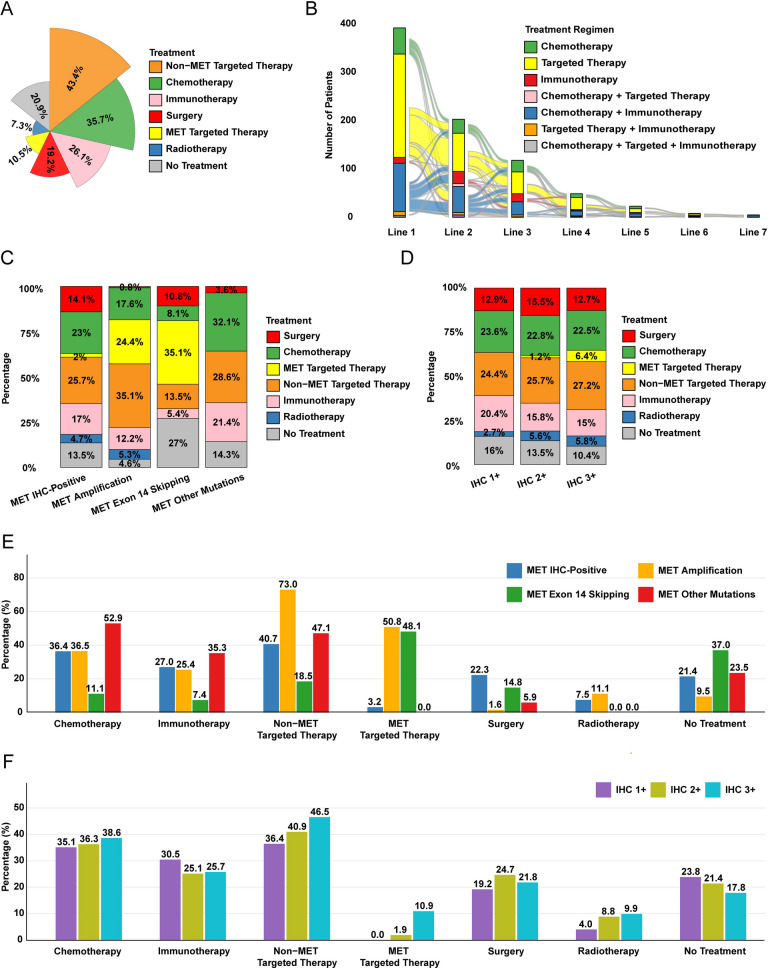
Treatment patterns across MET alteration subtypes. **A** Overall treatment regimen composition. **B** Sankey diagram showing sequential treatment evolution from first-line to seventh-line therapy. **C**-**D** Treatment regimen distribution across MET alteration subtypes (**C**) and IHC intensity groups (**D**); percentages represent the proportion of each treatment modality among total treatment frequencies. (**E**–**F**) Treatment acceptance rates across MET alteration subtypes (**E**) and IHC intensity groups (**F**); percentages represent the proportion of patients receiving each treatment among total patients in each group

In our cohort, 454 patients (79.1%) received anticancer therapy, yet 120 patients (20.9%) did not receive any treatment. Among them, treatment modalities included non-MET targeted therapy (*n* = 249, 43.4%), chemotherapy (*n* = 205, 35.7%), immunotherapy (*n* = 150, 26.1%), surgery (*n* = 110, 19.2%), MET targeted therapy (*n* = 60, 10.5%), and radiotherapy (*n* = 42, 7.3%) (Fig. 4A).

The Sankey diagram showed that targeted therapy was dominant across all treatment lines, accounting for 54.6% in Line 1st and 38.9% in Line 2nd. Chemotherapy plus immunotherapy was the second most common treatment modality (25.5% in Line 1st and 26.6% in Line 2nd). Chemotherapy alone also accounted for a certain proportion across treatment lines (13.8% in Line 1st, 14.3% in Line 2nd), while immunotherapy alone increased in Line 2 and beyond (3.1% in Line 1st, 12.3% in Line 2nd) (Fig. 4B).

#### Treatment patterns across MET alteration subtypes

Significant differences in treatment patterns existed among MET alteration subtypes. Targeted therapy predominated in MET Amplification (59.5%) and MET Exon 14 Skipping (48.6%) groups, while surgical treatment was more common in MET IHC-Positive (14.1%) and MET Exon 14 Skipping (10.8%) groups (Fig. 4C). Treatment patterns were similar across IHC intensity subgroups (Fig. 4D).

MET Amplification had the highest targeted therapy rate, with 73.0% receiving non-MET targeted therapy and 50.8% receiving MET targeted therapy. MET Other Mutations had the highest chemotherapy (52.9%) and immunotherapy (35.3%) rates, while the MET Exon 14 Skipping had the lowest (11.1% and 7.4%). MET Exon 14 Skipping also had the highest proportion of untreated patients (37%).

MET targeted therapy usage varied across subtypes. MET Amplification and MET Exon 14 Skipping had higher rates (50.8% and 48.1%), while MET IHC-Positive and MET Other Mutations had lower (3.2% and 0.0%). Among MET IHC-Positive subgroup, usage increased with IHC intensity (0.0% in IHC 1 +, 1.9% in IHC 2 +, 10.9% in IHC 3 +) (Fig. 4E-F).

### Characteristics of patients receiving MET-TKI

**Fig. 5:**
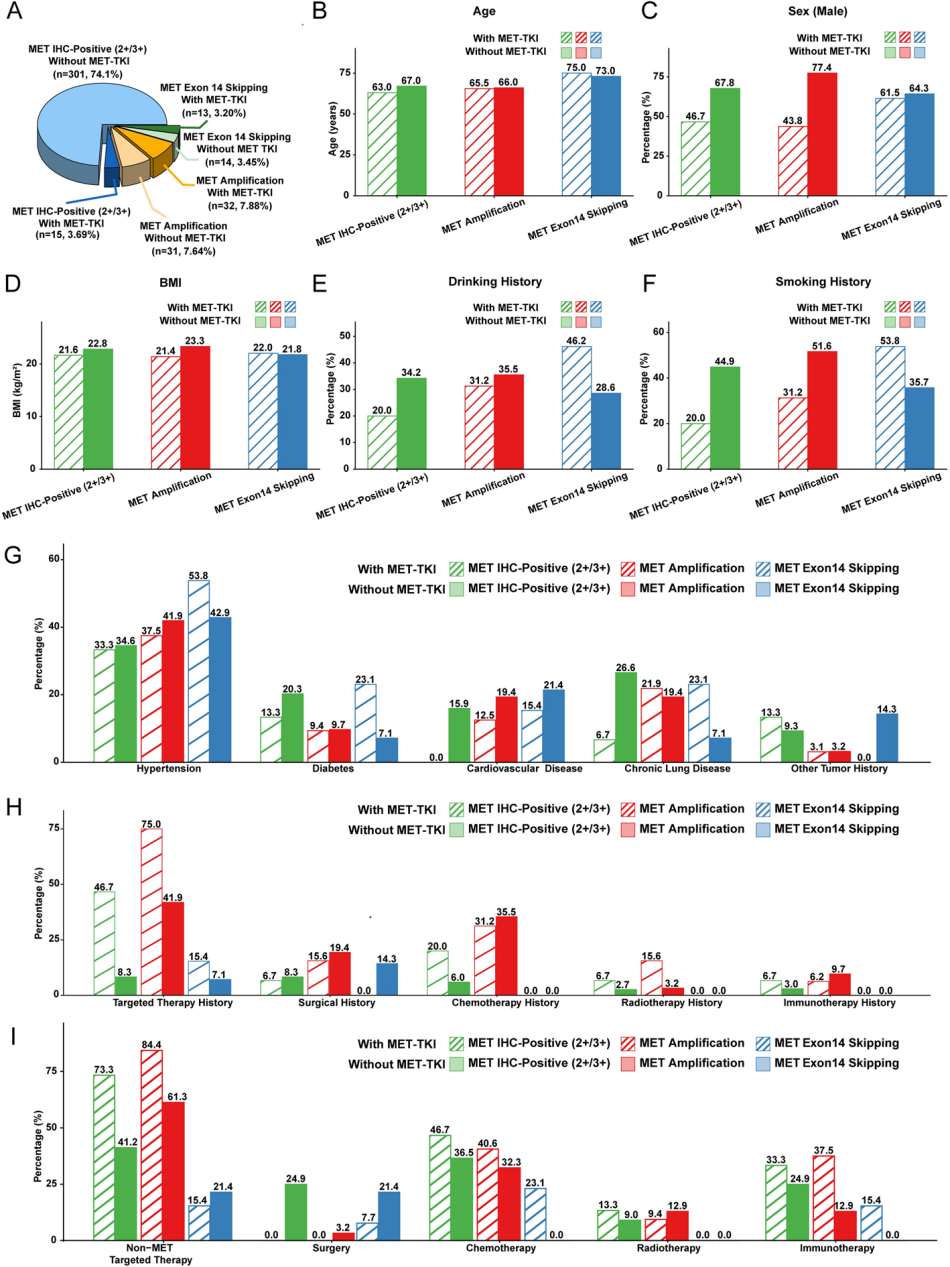
Comparison of patient characteristics by MET-TKI treatment status. **A** MET-TKI usage rates across MET alteration subgroups. (**B**-**F**) Comparison of age (**B**), gender (**C**), BMI (**D**), drinking history (**E**), smoking history (**F**), Comorbidity burden (**G**), prior anticancer treatment history (**H**), Comparison of treatment patterns (**I**). Hatched bars represent MET-TKI-treated group and solid bars represent untreated group

In clinical practice, MET-TKI is generally not recommended for MET IHC-Positive (1 +) or MET Other Mutations. None of these patients in our cohort received MET-TKI treatment. Therefore, subsequent MET-TKI analyses were conducted in 406 patients with MET IHC-Positive (2 +/3 +), MET Amplification, and MET Exon 14 Skipping. MET-TKI usage was highest in MET Amplification (50.8%), followed by MET Exon 14 Skipping (48.1%) and MET IHC-Positive (2 +/3 +) (4.75%) (Fig. 5A).

Baseline characteristics differed between MET-TKI-treated and untreated patients. MET-TKI-treated patients had a lower proportion of males across subgroups, most pronounced in MET Amplification (43.8% vs. 77.4%). Comorbidity profiles also varied, with MET Exon 14 Skipping showing higher rates of hypertension, diabetes, and chronic lung disease in treated patients (Fig. 5B-G).

MET-TKI-treated patients had substantially higher rates of prior targeted therapy across all subtypes (MET IHC-Positive (2 +/3 +): 46.7% vs. 8.3%; MET Amplification: 75.0% vs. 41.9%; MET Exon 14 Skipping: 15.4% vs. 7.1%) (Fig. 5H).

Regarding treatment patterns after MET detection, MET-TKI-treated patients had higher rates of chemotherapy and immunotherapy but lower rates of surgery. In MET IHC-Positive (2 +/3 +) and MET Amplification, treated patients had higher non-MET targeted therapy rates (73.3% vs. 41.2% and 84.4% vs. 61.3%, respectively), while this rate was lower in MET Exon 14 Skipping (15.4% vs. 21.4%) (Fig. 5I).

### Survival analysis

**Fig. 6:**
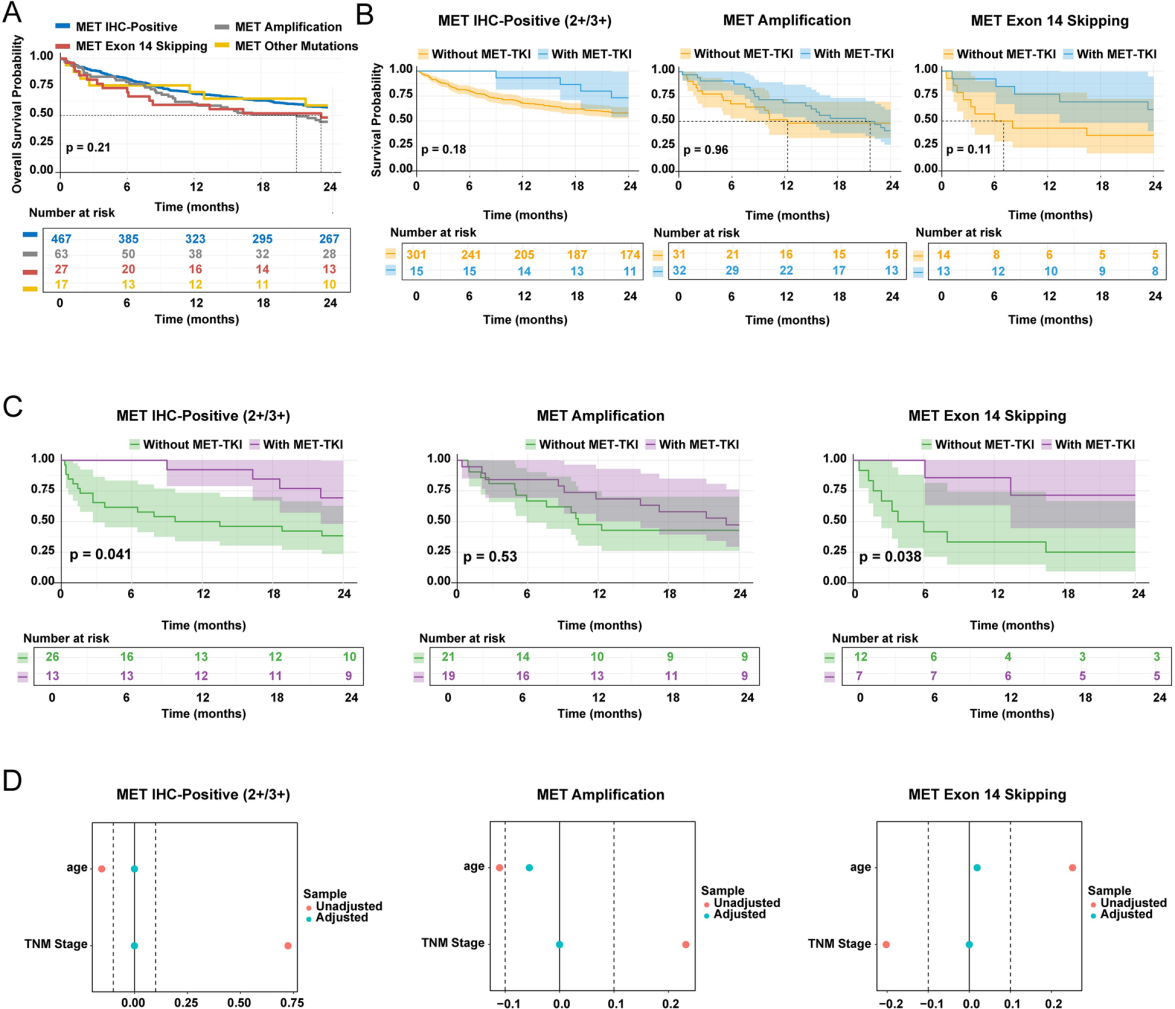
Survival analysis of MET alteration subtypes. **A** Kaplan–Meier curves comparing overall survival across MET alteration subtypes. **B** Overall survival comparison between MET-TKI-treated and untreated patients in MET IHC-Positive (2 +/3 +), MET Amplification, and MET Exon 14 Skipping before (**B**) and after (**C**) PSM. **D** SMD for age and TNM stage before and after PSM. All survival analyses censored at 24 months of follow-up; shaded regions represent 95% CI

The 24-month OS rates differed across MET alteration subtypes. Compared with MET IHC-Positive (57.2%) and MET Other Mutations (58.8%), MET Amplification (44.4%) and MET Exon 14 Skipping (48.1%) showed lower survival rates (Table 3) (Fig. 6A).

**Table 3:** Overall survival at 24 months by MET alteration subtypes and treatment strategy

MET Subgroup	Treatment	OS Rate (%)				*p*-value
		6-month	12-month	18-month	24-month	
Overall Cohortᵃ						
MET IHC-Positive (*n* = 467)		82.2 (78.8–85.8)	69.2 (65.1–73.5)	63.2 (58.9–67.7)	57.2 (52.9–61.8)	0.21
MET Amplification (*n* = 63)		79.4 (70.0–90.0)	60.3 (49.4–73.7)	50.8 (39.8–64.8)	44.4 (33.7–58.6)	
MET Exon 14 Skipping (*n* = 27)		70.4 (55.1–89.9)	59.3 (43.3–81.0)	51.9 (36.1–74.6)	48.1 (32.6–71.2)	
MET Other Mutation (*n* = 17)		76.5 (58.7–99.5)	70.6 (51.9–95.9)	64.7 (45.5–91.9)	58.8 (39.5–87.6)	
Before PSM^b^						
MET IHC-Positive (2 +/3 +) (*n* = 301/15)	Without MET-TKI	80.1 (75.7–84.7)	68.1 (63.0–73.6)	62.1 (56.9–67.9)	57.8 (52.5–63.7)	0.18
	With MET-TKI	100.0 (100.0–100.0)	93.3 (81.5–100)	86.7 (71.1–100.0)	73.3 (54.0–99.5)	
MET Amplification (*n* = 32/31)	Without MET-TKI	67.7 (53.1–86.4)	51.6 (36.7–72.6)	48.4 (33.6–69.6)	48.4 (33.6–69.6)	0.96
	With MET-TKI	90.6 (81.1–100.0)	68.8 (54.4–86.8)	53.1 (38.4–73.6)	40.6 (26.7–61.8)	
MET Exon 14 Skipping (*n* = 14/13)	Without MET-TKI	50.0 (29.6–84.4)	42.9 (23.4–78.5)	35.7 (17.7–72.1)	35.7 (17.7–72.1)	0.11
	With MET-TKI	92.3 (78.9–100.0)	76.9 (57.1–100.0)	69.2 (48.2–99.5)	61.5 (40.0–94.6)	
After PSM^c^						
MET IHC-Positive (2 +/3 +) (*n* = 26/13)	Without MET-TKI	61.5 (45.4–83.4)	50.0 (34.0–73.4)	46.2 (30.5–69.9)	38.5 (23.7–62.5)	0.041
	With MET-TKI	100.0 (100.0–100.0)	92.3 (78.9–100.0)	84.6 (67.1–100.0)	69.2 (48.2–99.5)	
MET Amplification (*n* = 21/19)	Without MET-TKI	66.7 (49.3–90.2)	47.6 (30.4–74.6)	42.9 (26.2–70.2)	42.9 (26.2–70.2)	0.53
	With MET-TKI	84.2 (69.3–100.0)	68.4 (50.4–92.9)	57.9 (39.5–85.0)	47.4 (29.5–76.1)	
MET Exon 14 Skipping (*n* = 12/7)	Without MET-TKI	41.7 (21.3–81.4)	33.3 (15.0–74.2)	25.0 (9.4–66.6)	25.0 (9.4–66.6)	0.038
	With MET-TKI	100.0 (100.0–100.0)	85.7 (63.3–100.0)	71.4 (44.7–100.0)	71.4 (44.7–100)	

*Abbreviations*: *OS* overall survival (date of tissue collection to last contact), *CI* confidence interval, *PSM* propensity score matching

ᵃOverall cohort not stratified by treatment (*n* = 574 total)

ᵇBefore PSM, sample sizes as (Without MET-TKI/With MET-TKI). MET IHC-Positive(1 +) excluded (*n* = 151, no targeted therapy); MET Other Mutation excluded (*n* = 17, no targeted therapy)

ᶜAfter 1:2 PSM adjusted for age and TNM stage. Sample sizes (Without MET-TKI/With MET-TKI) shown post-matching. All SMD < 0.1

All patients were censored at 24 months of follow-up

*P*-value from log-rank test

In terms of MET-TKI treatment response, all subgroups showed survival benefit trends. In MET IHC-Positive (2 +/3 +), the 24-month OS rate was 73.3% in treated patients vs. 57.8% in untreated patients. The difference was more pronounced in MET Exon 14 Skipping (61.5% vs. 35.7%). In MET Amplification, the 24-month OS rate was slightly higher in treated patients at 18 months (53.1% vs. 48.4%) but no sustained advantage was observed at 24 months (40.6% vs. 48.4%) (Table 3) (Fig. 6B).

To reduce baseline differences, propensity score matching (PSM) was performed adjusting for age and TNM stage. All covariates achieved SMD < 0.1 after matching, indicating good balance (Table 3) (Fig. 6C, D). Post-PSM, MET-TKI treatment was associated with survival benefit in MET IHC-Positive (2 +/3 +) patients (24-month OS: 69.2% vs. 38.5%, *P = *0.041). This trend persisted in MET Exon 14 Skipping (71.4% vs. 25.0%, *P = *0.038). In MET Amplification, however, survival difference was minimal (47.4% vs. 42.9%, *P = *0.53) (Fig. 6C). Given that MET Amplification may arise as resistance to prior EGFR-TKI therapy, an additional exploratory PSM analysis was performed in MET Amplification patients adjusting for prior EGFR-TKI therapy. After matching, a similar trend was observed (24-month OS: 38.1% vs. 33.3%, *P = *0.28) (Figure S1).

## Discussion

Based on real-world data from Southeastern China, this study described demographic, risk factor exposure, comorbidity burden, pathological characteristics, co-mutation landscapes, treatment patterns, and survival outcomes in NSCLC patients with MET alterations, revealing heterogeneity across subtypes and providing evidence for precision stratified management.

MET-altered patients in this cohort showed prominent elderly age (66–74 years), male predominance (58.8%–68.5%), and smoking enrichment (> 40%), contrasting sharply with classic driver genes such as EGFR and ALK, which are commonly found in younger, female, and non-smoking populations. Our findings were consistent with Israeli, Dutch, and US cohorts [13], as well as a multi-ancestry genomic study of over 70,000 patients by Miura [14]. Together, these data suggested a stable demographic profile of MET alterations across ethnicities.

The treatment patterns for MET-altered patients in southeastern China exhibited considerable complexity, with some patients receiving up to seven lines of therapy. Targeted therapy predominated across treatment lines. This pattern contrasted with cohorts from Italy (immunotherapy-predominant) [15] and India (chemotherapy-predominant) [16], reflecting geographic variations in treatment selection. MET-TKI accessibility improved following the approval of savolitinib in China [7] and its inclusion in the national reimbursement program as second-line therapy [17]. In our cohort, usage rates were 50.8% in patients with MET amplification and 48.1% in those with MET Exon 14 skipping. However, 20.9% of patients remained untreated, likely due to older age and high comorbidity burden [17, 18]. Economic conditions, drug accessibility, and patient baseline characteristics collectively shaped the distinct treatment patterns in southeastern China.

Although most patients received aggressive multi-line therapy, overall prognosis among MET-altered patients remained poor, with 24-month OS rates of 48.1% for MET Exon 14 skipping and 44.4% for MET amplification. These findings are consistent with the 24-month OS rate of 48% reported in the VISION study [5]. Within this overall landscape, we further investigated heterogeneity across MET alteration subtypes.

MET amplification exhibited the most aggressive phenotype. In our cohort, this subtype was enriched for TP53 and EGFR co-mutations. TP53 mutations are associated with genomic instability and enhanced tumor cell adaptability [19]. EGFR co-mutations suggested that MET amplification may have arisen as a bypass mechanism following EGFR-TKI therapy [20, 21], which aligned with our finding that over half of patients had received prior targeted therapy. This subtype also showed a high prevalence of bone metastasis (36.5%) and brain metastasis (17.5%). Together, these molecular and clinical characteristics might contribute to the inferior prognosis and limited response to MET-TKI in this subtype.

MET Exon 14 skipping showed a distinct driver phenotype with clear MET-TKI benefit. The co-mutation profile was dispersed without predominant drivers, consistent with the driver gene mutual exclusivity reported before [22]. Patients with MET Exon 14 skipping received less chemotherapy and immunotherapy, while MET-TKI usage was higher (48.1%), reflecting clinical preference for targeted therapy. This aligns with evidence that MET signaling may confer chemotherapy resistance [23] and immunotherapy offers limited benefit [24–26]. After PSM, MET-TKI was associated with significantly higher 24-month OS versus no MET-TKI treatment (71.4% vs 25.0%). These results are consistent with GEOMETRY mono-1 [27] and VISION [5], and support NCCN [28] and ASCO [29] guidelines recommending MET-TKI as first-line therapy.

MET IHC-Positive patients comprised the largest subgroup in our cohort (81.4%), yet its clinical significance remained debated. Our findings suggested that MET IHC-Positive (2 +/3 +) may represent a distinct subtype. Adenocarcinoma prevalence increased with IHC intensity (57.6% in IHC 1 +, 73.5% in IHC 2 +, and 87.1% in IHC 3 +, *p* < 0.05). This was consistent with the findings of Zhan et al. in a Chinese population [30], supporting a link between MET overexpression and glandular differentiation. Patients with MET IHC-Positive (2 +/3 +) showed a trend toward MET-TKI benefit. Recent studies, including Teliso-V, have also demonstrated significant efficacy of MET-targeted therapy in MET Overexpression [11, 31]. In NGS-limited settings, IHC offers a cost-effective screening approach to identify patients potentially benefiting from MET-targeted therapy.

However, this study has several limitations. First, the single-center retrospective design may limit generalizability due to geographic restrictions. Second, the relatively small sample size limited statistical power. Third, we were unable to definitively distinguish between de novo and acquired MET amplification due to lack of baseline MET testing in some patients. Finally, long-term survival outcomes for some patients remain under follow-up. Future multi-center, large-scale studies with comprehensive baseline molecular profiling are needed to clarify the MET-altered NSCLC landscape and define optimal treatment strategies across subtypes.

## Conclusions

This study characterizes the comprehensive landscape of MET-altered NSCLC in southeastern China. MET alterations occur predominantly in the elderly, males, and those with smoking history. MET Exon 14 skipping shows driver-dependent characteristics with clear MET-TKI benefit. MET Amplification presents the most aggressive phenotype. MET IHC-Positive predominates (> 80%) with heterogeneity and MET IHC-Positive(2 +/3 +) may benefit from MET-TKI. These findings inform subtype-based management of MET-altered NSCLC in southeastern China. Future multicenter, large-scale studies are warranted to further clarify clinical characteristics and optimize treatment strategies across subtypes.

## Supplementary Information

Supplementary Material 1. Table S1. Lesion location and biopsy method across MET alteration subtypes. Table S2. Lesion location and biopsy method according to IHC intensity in MET IHC-Positive patients. Table S3. Histological type distribution across MET IHC-Positive levels. Figure S1. Survival analysis in MET Amplification after adjusting for prior EGFR-TKI therapy. (A) Kaplan-Meier curves after PSM adjusting for TNM stage and EGFR-TKI therapy history. (B) SMD before and after PSM. Survival censored at 24 months; shaded regions represent 95% CI.

## Data Availability

The datasets used and/or analysed during the current study are available from the corresponding author on reasonable request.

## Abbreviations

MET: Mesenchymal-epithelial transition factor
NSCLC: Non-small cell lung cancer
IHC: Immunohistochemistry
TKI: Tyrosine kinase inhibitor
OS: Overall survival
PSM: Propensity score matching
RT-qPCR: Real-Time Quantitative Polymerase Chain
NGS: Next-Generation Sequencing
FISH: Fluorescence In Situ Hybridization
AJCC: American Joint Committee on Cancer
TNM: Tumor-node-metastasis
IQR: Interquartile range
SMD: Standardized mean difference
BMI: Body mass index
NCCN: National Comprehensive Cancer Network
ASCO: American Society of Clinical Oncology
