# Supplementary material for: The landscape of MET alterations in non-small cell lung cancer in Southeastern China: a real-world study
Source: BMC Cancer. 2026 May 26;26:883. doi: 10.1186/s12885-026-16211-y (PMC13393253; doi:10.1186/s12885-026-16211-y)
Supplement: Supplementary file 1 — Supplementary Material 1. Table S1. Lesion location and biopsy method across MET alteration subtypes. Table S2. Lesion location and biopsy method according to IHC intensity in MET IHC-Positive patients. Table S3. Histological type distribution across MET IHC-Positive levels. Figure S1. Survival analysis in MET Amplification after adjusting for prior EGFR-TKI therapy. (A) Kaplan-Meier curves after PSM adjusting for TNM stage and EGFR-TKI therapy history. (B) SMD before and after PSM. Survival censored at 24 months; shaded regions represent 95% CI. [file 12885_2026_16211_MOESM1_ESM.docx]

**Table S1. Lesion location and biopsy method across MET alteration subtypes.**

| Patients' characteristics | MET IHC-Positive (n=467) | MET Amplification (n=63) | MET Exon 14 Skipping (n=27) | MET Other Mutations (n=17) |
| --- | --- | --- | --- | --- |
| Lesion location, n (%) |  |  |  |  |
| Right lung | 219 (46.9%) | 28 (44.4%) | 16 (59.3%) | 7 (41.2%) |
| Left lung | 147 (31.5%) | 27 (42.9%) | 9 (33.3%) | 4 (23.5%) |
| Bilateral | 96 (20.6%) | 8 (12.7%) | 2 (7.4%) | 6 (35.3%) |
| Other | 1 (0.2%) | 0 (0.0%) | 0 (0.0%) | 0 (0.0%) |
| Biopsy method, n (%) |  |  |  |  |
| Lung puncture | 134 (28.7%) | 26 (41.3%) | 9 (33.3%) | 2 (11.8%) |
| Bronchoscopy | 143 (30.6%) | 19 (30.2%) | 9 (33.3%) | 10 (58.8%) |
| Lobectomy specimen | 104 (22.3%) | 6 (9.5%) | 5 (18.5%) | 4 (23.5%) |
| Lymph node puncture | 16 (3.4%) | 3 (4.8%) | 1 (3.7%) | 1 (5.9%) |
| Pleural effusion | 38 (8.1%) | 3 (4.8%) | 1 (3.7%) | 0 (0.0%) |
| Other | 14 (3.0%) | 2 (3.2%) | 1 (3.7%) | 0 (0.0%) |
| Multiple methods | 18 (3.9%) | 4 (6.3%) | 1 (3.7%) | 0 (0.0%) |

**Table S2.** **Lesion location and biopsy method according to IHC intensity in MET IHC-Positive patients.**

| Patients' characteristics | IHC 1+ (n=151) | IHC 2+ (n=215) | IHC 3+ (n=101) |
| --- | --- | --- | --- |
| Lesion location, n (%) |  |  |  |
| Right lung | 72 (47.7%) | 96 (44.7%) | 51 (50.5%) |
| Left lung | 53 (35.1%) | 63 (29.3%) | 31 (30.7%) |
| Bilateral | 23 (15.2%) | 54 (25.1%) | 19 (18.8%) |
| Other | 1 (0.7%) | 0 (0.0%) | 0 (0.0%) |
| Biopsy method, n (%) |  |  |  |
| Lung puncture | 37 (24.5%) | 62 (28.8%) | 35 (34.7%) |
| Bronchoscopy | 54 (35.8%) | 62 (28.8%) | 27 (26.7%) |
| Lobectomy specimen | 31 (20.5%) | 50 (23.3%) | 23 (22.8%) |
| Lymph node puncture | 3 (2.0%) | 6 (2.8%) | 7 (6.9%) |
| Pleural effusion | 10 (6.6%) | 26 (12.1%) | 2 (2.0%) |
| Other | 3 (2.0%) | 5 (2.3%) | 6 (5.9%) |
| Multiple methods | 13 (8.6%) | 4 (1.9%) | 1 (1.0%) |

**Table S3. Histological type distribution across MET IHC-Positive levels.**

|  | Non-adenocarcinoma,  n(%) | Adenocarcinoma,  n(%) | Chi square | P value |
| --- | --- | --- | --- | --- |
| Overall (n=467) | 134 (28.7%) | 333 (71.3%) | 26.7 | <0.001 |
| MET IHC-Positive Level |  |  |  |  |
| IHC 1+ (n=151) | 64 (42.4%) | 87 (57.6%) |  |  |
| IHC 2+ (n=215) | 57 (26.5%) | 158 (73.5%) |  |  |
| IHC 3+ (n=101) | 13 (12.9%) | 88 (87.1%) |  |  |
| Post-hoc Pairwise Comparisons* |  |  |  |  |
| IHC 1+ vs IHC 2+ |  |  | 9.39 | 0.007 |
| IHC 1+ vs IHC 3+ |  |  | 23.5 | <0.001 |
| IHC 2+ vs IHC 3+ |  |  | 6.64 | 0.030 |

P values adjusted using Bonferroni correction for multiple comparisons.


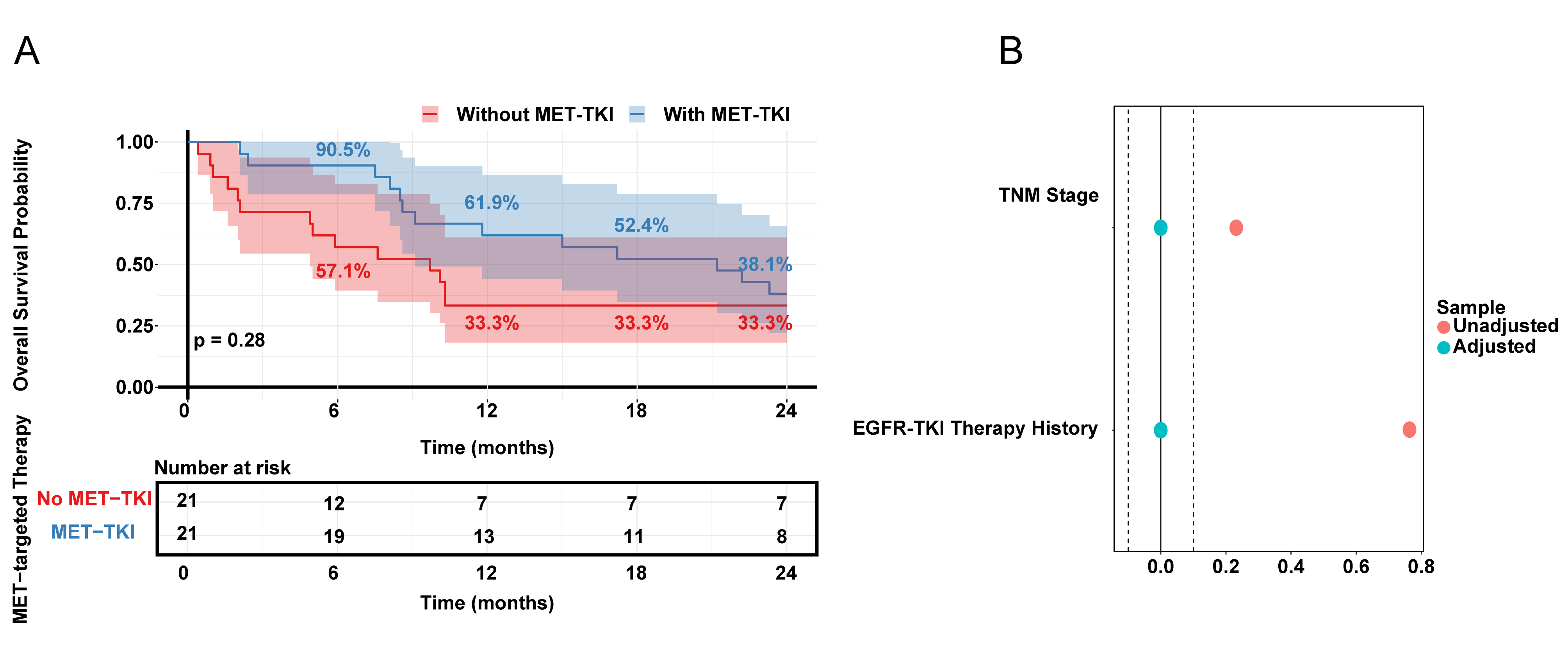


**Figure S1. Survival analysis in MET Amplification after adjusting for prior EGFR-TKI therapy.** (A) Kaplan-Meier curves after PSM adjusting for TNM stage and EGFR-TKI therapy history. (B) SMD before and after PSM. Survival censored at 24 months; shaded regions represent 95% CI.
